# Total Iliocaval Reconstruction in a Complex Palliative Patient with Malignant Inferior Vena Cava Syndrome

**DOI:** 10.3390/curroncol31070294

**Published:** 2024-07-09

**Authors:** Jack Lofroth, Amir Pourghadiri, Anastasia Hadjivassiliou, Manraj Heran, Gerald Legiehn, Stephen Ho, Ravjot Dhatt

**Affiliations:** 1 Faculty of Medicine, University of British Columbia, Vancouver, BC V6T 1Z4, Canada; 2 Department of Radiology, Vancouver General Hospital, Vancouver, BC V5Z 1M9, Canada

**Keywords:** inferior vena cava syndrome, iliocaval reconstruction, metastatic lung cancer, palliative

## Abstract

Inferior vena cava (IVC) compression secondary to mass effect is accompanied by edema, ascites, back and abdominal pain, and central nervous system symptoms. Most IVC syndrome cases described in the literature focus on the focal treatment of IVC lesions, and reports of complete iliocaval reconstructions secondary to malignant IVC syndrome in the palliative context are limited. In this case report, we describe the clinical presentation, technical approach, and symptomatic outcomes of a patient with extensive malignant compression and invasion of the iliofemoral venous system. An 82-year-old male with metastatic lung cancer invading the right upper quadrant of the abdomen presented with scrotal and bilateral lower extremity edema, as well as anasarca. Computed tomography (CT) demonstrated an 11 cm right adrenal metastasis and extensive retroperitoneal lymphadenopathy resulting in the compression of the IVC and iliac veins. Femoral venography demonstrated extensive collateral venous pathway formation with the opacification of the para-lumbar and vertebral veins, in addition to the vertebral/sacral venous plexus. Iliocaval reconstruction was performed using venous-dedicated stents. This case report highlights a technically successful total iliocaval reconstruction in a complex palliative patient with diffuse metastatic disease resulting in IVC compression and syndrome.

## 1. Introduction

Inferior vena cava syndrome (IVCS) is a rare and under-reported entity caused by mass effect, agenesis, hypoplasia, stenosis, and/or thrombosis [1]. The exact incidence and prevalence of IVCS are unknown; however, IVCS is associated with metastatic disease, polycystic liver/kidney disease, retroperitoneal fibrosis secondary to pelvic irradiation, and primary tumors such as leiomyosarcoma [1]. The clinical presentation of IVCS results from a venous outflow obstruction, leading to a constellation of symptoms including anasarca and ascites, as well as lower-extremity and genital edema. Initially, symptoms related to obstruction can be suppressed due to pelvic, abdominal, and paravertebral collateral venous formation [2].

Anticoagulation therapy may alleviate lower-extremity edema symptoms but does not address the underlying pathology of venous outflow obstruction [3]. Endovascular stent placement is one strategy to manage malignant and non-malignant IVCS, which has demonstrated a rapid and effective therapeutic response [4,5].

Comprehensive reporting on the technical or clinical success of malignant IVCS causing extensive venous compression is limited, particularly in the palliative population. However, the available studies suggest that iliocaval reconstruction is a safe, viable, and minimally invasive option that provides symptomatic relief and low procedural complication rates in the palliative setting [6,7,8,9]. This case report highlights a complex palliative presentation of malignant inferior vena cava syndrome (IVCS) managed with complete iliocaval reconstruction.

## 2. Detailed Case Description

**Figure 1:**
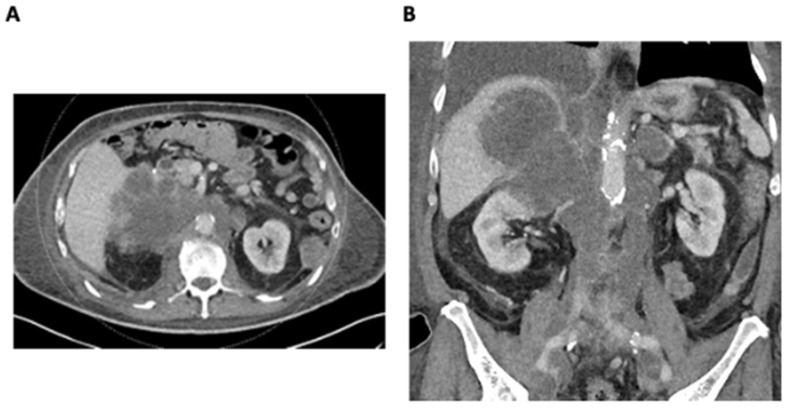
(**A**) Axial CT demonstrating a large infiltrative right adrenal metastasis and retroperitoneal lymphadenopathy. (**B**) Coronal CT demonstrating a large infiltrative right adrenal metastasis and retroperitoneal lymphadenopathy resulting in the compression and thrombosis of the IVC and iliac veins.

An 82-year-old male with a significant family history of malignancy and the BRCA2 functional variant detected in metastatic lung cancer presented with a rapidly enlarging right adrenal metastasis measuring up to 11 cm, invading the right liver lobe and infiltrating the upper pole of the right kidney. There was also extensive retroperitoneal lymphadenopathy, resulting in severe IVC and iliac vein compression and thrombosis (Figure 1A,B). A 3 cm segment of infrahepatic IVC below the hepatic vein confluence was patent. There was evidence of new left adrenal metastasis, the worsening of osteoblastic metastases, and recurrent pleural and bibasilar pulmonary metastases. Disease progression resulted in severe scrotal and bilateral lower-extremity edema, ascites, body wall edema, and dyspnea.

**Figure 2:**
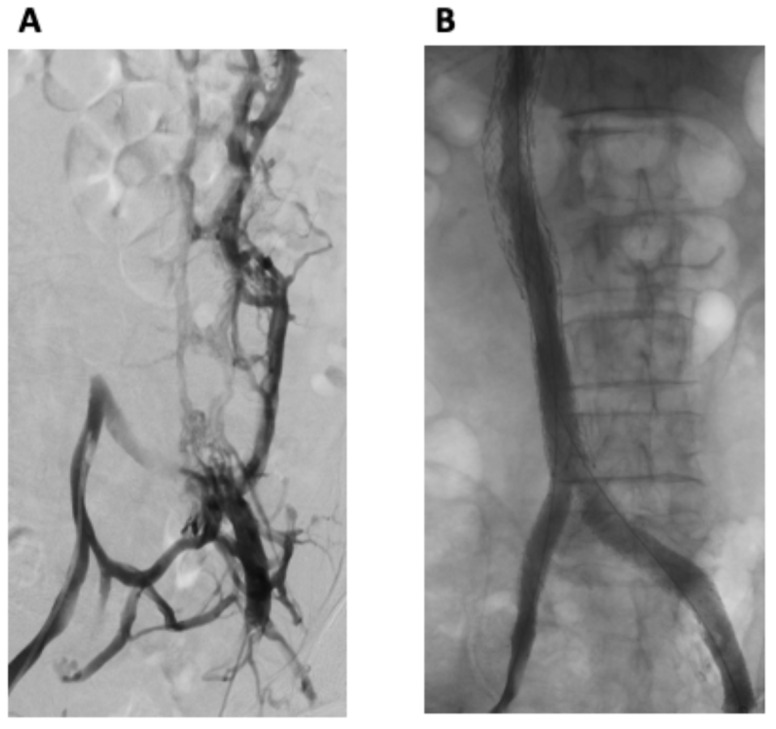
(**A**) Bilateral femoral venography demonstrated an occluded IVC with extensive collateral venous pathways, with the opacification of the paralumbar and intervertebral veins as well as the vertebral/sacral venous plexus, including the epidural veins within the spinal canal. (**B**) Post-intervention venography from both groins demonstrated widely patent stents with successful iliac and IVC reconstruction and no residual collateral flow.

The procedure was performed using a general anesthetic technique. Bilateral common femoral vein (CFV) and right internal jugular vein (RIJV) micropuncture access was obtained. Bilateral femoral venography demonstrated an occluded IVC with extensive collateral venous pathways, with the opacification of the paralumbar intervertebral veins as well as the vertebral/sacral venous plexus, including the epidural veins within the spinal canal (Figure 2A). Drainage to the heart was via the hemi-azygous system. Large left-sided pelvic collaterals drained into the right internal iliac vein.

A 100 cm 5 Fr AR3 guide catheter and a 135 cm 4 Fr Navicross were used with a 260 cm Advantage Glidewire to manipulate through the occluded IVC to the infrahepatic IVC. The guidewire was snared and brought out through the jugular access. This was performed bilaterally from both femoral accesses. Intravascular ultrasound (IVUS) was performed and demonstrated the complete occlusion of the IVC from below the level of the iliac bifurcation to the intrahepatic IVC likely secondary to the tumor and bland thrombus, with superimposed extrinsic compression from the extensive infiltrative abdominal metastases.

Following the pre-dilation of the IVC with a 12 × 40 mm Atlas balloon, iliac and IVC reconstruction was performed. Initially, from the right groin access, an 18 × 120 mm Medtronic Abre venous self-expanding stent was inserted into the upper IVC, below the confluence of the hepatic veins and IVC. Subsequently, two overlapping 16 × 100 mm Medtronic Abre venous self-expanding stents were inserted with the caudal aspect just above the iliac bifurcation. Balloon dilation of the IVC stents was performed with 14 and 16 mm Atlas balloons, respectively.

Attention was then directed toward the iliac reconstruction with 14 mm Cook Zilver Vena self-expanding stents (COOK MEDICAL LLC, Bloomington, IN, USA). The stents were placed with the cephalad aspect of both stents touching at the iliac bifurcation. Subsequently, the entirety of the stented IVC was angioplastied with a 18 × 40 mm Atlas balloon and the reconstructed iliac veins were angioplastied with a 16 × 100 mm Atlas balloon.

Venography from both groins demonstrated widely patent stents with successful iliac and IVC reconstruction and no residual collateral flow (Figure 2B). Antegrade flow of contrast through the stented iliac and IVC components to the right heart was demonstrated. The patient tolerated the procedure well, with no immediate complications.

## 3. Discussion

Inferior vena cava reconstruction is a complex procedure that requires the careful consideration of the technical success and clinical benefit offered for palliative patients with a significant tumor burden. To the best of our knowledge, the endovascular management of IVC syndrome was first described in the late 1980s using Gianturco expandable metallic stents in an 82-year-old patient presenting with abdominal and bilateral lower-extremity edema secondary to radiation-induced retroperitoneal fibrosis [10]. Since this period, there have been significant advancements in diagnostic imaging modalities, technical expertise, stent technology, and pharmacologic therapies.

Diagnosis and endovascular preoperative treatment planning are performed with the assistance of various imaging modalities. Doppler ultrasound provides information regarding potential access flows, the intraluminal thrombus, and the flow characteristics [1]. However, Doppler ultrasound is operator-dependent and limited by the patient’s body characteristics, including bowel gas, adiposity, the distortion of vascular structures due to neoplastic disease, and intra-abdominal ascites. Therefore, cross-sectional imaging using CT or MR venography remains the mainstay for the diagnosis and mapping of the vascular anatomy, extent of disease, and intravascular thrombus.

Intraprocedurally, intravascular ultrasound (IVUS) is gaining traction for a wide variety of disease pathologies, including venous reconstruction. The advantages of IVUS include the dynamic assessment of the vascular structures, accurate measurements of the vascular disease severity allowing for appropriate stent sizing, a reduction in procedural time, and the decreased use of contrast and radiation [11]. As the first prospective study comparing IVUS and conventional multiplanar venography (CMPV) in iliofemoral venous obstructions, the VIDIO trial revealed that the adjunctive use of IVUS provided greater sensitivity in detecting stenotic lesions and an altered treatment course in 57% of participants [11]. Despite the advantages of IVUS, its utility is site-dependent, and barriers to its use include the cost, operator comfort, access, and further evidence required to support its application [12].

In addition to the diagnostic value of CT/MR venography and IVUS, these modalities also guide the optimal selection of the endovascular stent length and diameter. Careful consideration of the stent sizing is crucial as oversized stent diameters increase the risk of compression, whereas undersized stents increase the risk of migration [3]. At our institution, IVUS is used routinely as an adjunctive modality for venous reconstruction. In our approach, the cephalad and caudal extent of the stents are placed into non-compressed normal caliber veins, with the stent diameter measured 1–2 mm above the normal vein caliber to prevent stent migration.

The physical profiles of modern-day dedicated venous stents have demonstrated impressive durability, with high crush resistance and radial strength, resulting in 12-month patency of 88% [13]. The use of covered and bare stents has not been directly compared in IVC syndrome. However, retrospective studies examining covered and uncovered stents in superior vena cava (SVC) syndrome have demonstrated comparable clinical and technical success but superior long-term patency for covered stents [14,15]. In the setting of extensive iliofemoral disease, we overlap flexible and high-radial-strength self-expanding venous stents with bifurcated stents extending into the iliac veins to minimize the risk of stent fracture and allow for optimal expansion and conformability to the iliac vessels.

In the periprocedural phase, interventionists may be faced with challenging access sites and insufficient venodilation, which can be mitigated by careful preoperative planning and the appropriate measurement and selection of the stent calibers, respectively [16]. In addition, considering the extent of the disease and multiple access points (e.g., CFV, RIJV, and bilateral great saphenous vein) in the preprocedural phase will help to mitigate the aforementioned challenges [17]. Another periprocedural complication is puncture-site hematoma, which is typically self-limited and can be managed with manual compression or pressure-assisted devices [16]. Lastly, a concerning but rare complication is pulmonary edema [18]. To reduce the risk of this event, our patients are screened with pre- and post-procedural echocardiograms for the assessment of the right heart function and ejection fraction, and they are monitored in the intensive care unit 24–48 h post-intervention. In cases of ongoing or progressing pulmonary edema, conservative therapies such as diuretics and, if necessary, vasopressor support are utilized.

Follow-up for IVC obstruction typically involves daily clinical examinations post-procedure, with adjunctive imaging if there are concerns about postprocedural complications, including in-stent stenosis, thrombus formation, pulmonary embolism, or nephrotic syndrome resulting from renal vein compression. In patients with a baseline hypercoagulable state, thrombus formation in the IVC or renal veins may lead to nephrotic-range proteinuria [19]. Therefore, imaging follow-up can be performed with duplex ultrasound and CTV/MRV to monitor for these potential complications. For patients with life expectancies exceeding greater than a month, CT/MRI follow-up is performed at one month and subsequently at 6-month intervals to assess stent patency. Studies generally evaluate stent patency at multiple intervals in the first year, followed by annual surveillance thereafter [20,21,22].

Anticoagulation therapy after venous stenting is a topic of ongoing debate with a paucity of data. A recent expert panel review article acknowledges that anticoagulant therapy after venous stenting is crucial in preventing thrombotic events and maintaining long-term stent patency [23]. However, the current guidelines have not demonstrated the superior performance of specific agents or combination therapies [24]. At our institution, patients are fully heparinized during the procedure with an ACT range of 250–300. After achieving hemostasis, patients are transitioned to unfractionated heparin with a target PTT range of 65–90 for 24–48 h and a vitamin K antagonist to allow for reversal. Additionally, we place patients on acetylsalicylic acid (ASA) 81 mg. All anticoagulation decisions are made in consultation with our hematology colleagues. Ultimately, the underlying hypercoagulable states must be balanced with the risk of internal bleeding and should be assessed on a case-by-case basis.

An overarching goal in endovascular interventions, particularly in palliative patients with a greater risk burden, includes considering the technical success rate. Technical success is commonly described as the successful placement of venous stents, the restoration of the vein diameter, and the absence of collateral flow [8,25,26,27]. Percutaneous stent placement has demonstrated high technical success rates with rapid and sustained symptom relief for IVCS; however, most cases in the literature have described the treatment of focal benign and malignant IVC lesions [3,25,28]. The reported technical success rates for non-malignant and malignant IVC reconstruction with or without iliac or iliofemoral obstruction range from 78 to 100% [29,30,31]. A systematic review found that the technical success rates for malignant IVC reconstruction ranged between 94 and 100% (31). In our case, technical success was verified via post-procedural venography, demonstrating a widely patent iliocaval venous system with no evidence of residual collateral flow.

Another important consideration is the cost and affordability of iliocaval reconstruction. Our center, based in Canada, benefits from the majority of interventional procedures being covered by a public healthcare system, rendering estimations of costs challenging. To the best of our knowledge, one conference abstract has addressed this issue, concluding that the majority of the costs for iliocaval reconstruction are driven by the procedure’s duration, with an estimated cost of USD 21,159.64 [32]. One of the main contributors to the increased costs was the duration of anesthesiology staff presence, with costs ranging from USD 20,031.53 to 21,049.71. However, further detailed reports are required to gain a greater understanding of the contributing factors, including consumables and staff wages. With advancements in stent technology and interventional procedures, the efficiency is expected to improve, hence reducing the duration and cost of iliocaval reconstruction.

Ultimately, the clinical benefit and therapeutic relief of symptoms are paramount from the patient’s perspective. Clinical improvements for non-malignant and malignant IVC reconstruction include the alleviation of ascites, edema, and anasarca. Symptomatic relief in patients with non-malignant obstruction has been shown to be more readily achieved compared to those with malignant obstruction [3,26]. Augustin et al. (2022) demonstrated primary patency of 93% with a median follow-up of 65 days and a significant improvement in lower-extremity edema in patients with malignancy-related IVCS [7]. A recent retrospective analysis of 37 patients with malignant IVCS found that 78% of patients reported a significant improvement in clinical symptoms following IVC reconstruction compared to pre-procedural symptoms [8]. Data on the expected duration of symptomatic alleviation for malignant IVCS reconstruction are scarce, largely due to the variability in patient survival times post-procedure. However, recent evidence demonstrated stent patency rates of 93% at 1 month, 81% at 3 months, and 69% at 6 months, as well as an overall 21% prevalence of stent occlusion after iliocaval reconstruction [8]. The most common cause of stent occlusion was external compression from the tumor. Secondary stent patency requiring re-intervention has been reported at 91.5%, ranging from 77% to 100% [31].

Clinically, our patient demonstrated an improvement 24–48 h post-procedure, with no symptom recurrence. Physical examination on day four revealed the recovery of scrotal and bilateral lower-extremity edema and the patient-reported alleviation of pain with a reduction in analgesic requirements. Unfortunately, no radiological follow-up was available due to the patient’s expected short life expectancy. Daily clinical follow-up was performed until the patient’s demise 3 weeks after the procedure from malignancy-related complications.

## 4. Conclusions

Iliocaval reconstructions in the setting of extensive IVC and iliac vein compression and/or occlusion secondary to diffuse metastatic disease have scarcely been described as these procedures carry greater risks and technical challenges. This case demonstrated a technically successful complete iliocaval reconstruction in a palliative patient suffering from IVCS due to advanced metastatic disease. This case and our growing institutional experience have demonstrated that the restoration of iliocaval systems provides symptomatic relief and improves the quality of life in patients with complex metastatic disease.

## Data Availability

No new data were created or analyzed in this case report, and therefore data sharing is not applicable.
